# Gene Expression of *CSF3R*/CD114 Is Associated with Poorer Patient Survival in Glioma

**DOI:** 10.3390/ijms25053020

**Published:** 2024-03-05

**Authors:** Samir Ale Bark, Matheus Dalmolin, Osvaldo Malafaia, Rafael Roesler, Marcelo A. C. Fernandes, Gustavo R. Isolan

**Affiliations:** 1 Graduate Program in Principles of Surgery, Mackenzie Evangelical University, Curitiba 80730-000, PR, Brazil; 2 The Center for Advanced Neurology and Neurosurgery (CEANNE), Porto Alegre 90560-010, RS, Brazil; 3 InovAI Lab, nPITI/IMD, Federal University of Rio Grande do Norte, Natal 59078-970, RN, Brazil; matheusdalmolinrs@gmail.com (M.D.); mfernandes@dca.ufm.br (M.A.C.F.); 4 Bioinformatics Multidisciplinary Environment (BioME), Federal University of Rio Grande do Norte, Natal 59078-970, RN, Brazil; 5 Department of Pharmacology, Institute for Basic Health Sciences, Federal University of Rio Grande do Sul, Porto Alegre 90035-003, RS, Brazil; 6 Cancer and Neurobiology Laboratory, Experimental Research Center, Clinical Hospital (CPE-HCPA), Federal University of Rio Grande do Sul, Porto Alegre 90035-003, RS, Brazil; 7 National Science and Technology Institute for Children’s Cancer Biology and Pediatric Oncology—INCT BioOncoPed, Porto Alegre 90035-003, RS, Brazil; 8 Department of Computer Engineering and Automation, Federal University of Rio Grande do Norte, Natal 59078-970, RN, Brazil

**Keywords:** *CSF3R*, CD114, granulocyte colony stimulating factor, glioma, glioblastoma, brain tumor

## Abstract

Gliomas comprise most cases of central nervous system (CNS) tumors. Gliomas afflict both adults and children, and glioblastoma (GBM) in adults represents the clinically most important type of malignant brain cancer, with a very poor prognosis. The cell surface glycoprotein CD114, which is encoded by the *CSF3R* gene, acts as the receptor for the granulocyte colony stimulating factor (GCSF), and is thus also called GCSFR or CSFR. CD114 is a marker of cancer stem cells (CSCs), and its expression has been reported in several cancer types. In addition, CD114 may represent one among various cases where brain tumors hijack molecular mechanisms involved in neuronal survival and synaptic plasticity. Here, we describe *CSF3R* mRNA expression in human gliomas and their association with patient prognosis as assessed by overall survival (OS). We found that the levels of *CSF3R*/CD114 transcripts are higher in a few different types of gliomas, namely astrocytoma, pilocytic astrocytoma, and GBM, in comparison to non-tumoral neural tissue. We also observed that higher expression of *CSF3R*/CD114 in gliomas is associated with poorer outcome as measured by a shorter OS. Our findings provide early evidence suggesting that *CSF3R*/CD114 shows a potential role as a prognosis marker of OS in patients with GBM.

## 1. Introduction

Gliomas comprise about 80 percent of central nervous system (CNS) cancers in adults. In children, CNS cancers including pediatric gliomas represent the majority of solid tumors. Gliomas are classified into different types, namely astrocytoma, oligodendroglioma and glioblastoma (GBM). The most prevalent and lethal glioma type is GBM. Patients with this tumor type have a poor prognosis even after undergoing multimodal therapy combining surgical resection, radiotherapy, and treatment with temozolomide. Status of the isocitrate dehydrogenase (*IDH*) gene allows the classification of GBM into three groups, namely *IDH* wild-type GBM, which represents about 90% of cases, mutated *IDH*, or not specified GBM (NOS, unevaluated status) [1,2,3,4,5].

The cell surface protein CD114, encoded by the *CSF3R* gene, is a receptor for the granulocyte colony stimulating factor (GCSF), being thus also called GCSFR or CSFR [6,7]. Upon stimulation by GCSF, CD114 activates the transcription factor signal transducer and activator of transcription 3 (STAT3), which promotes a cancer stem cell (CSC) phenotype [8]. CD114 has been proposed as a marker for CSCs in neural crest-derived tumors such as neuroblastoma (NB) and melanoma [7,9,10]. Expression of CD114 is found in solid tumors including brain, ovarian, cervical, bladder, and skin cancers [7,10,11,12,13,14,15,16,17,18]. In addition, *CSF3R* mutations have been found in rare types of leukemia [19,20].

Brain cancer hijacks molecular and cellular mechanisms of neuronal plasticity [21,22,23,24,25,26]. GCSF acts as a growth factor stimulating the survival and plasticity of neurons and neural stem cells [27]. GCSF stimulates neuronal survival and neurogenesis [6] and acts synergistically with stem cell factor (SCF) to stimulate neurite outgrowth in cortical neurons [28]. The combination of GCSF and SCF also protects from neurodegeneration and promotes neurostructure network reorganization in a mouse model of traumatic brain injury [29]. Systemic administration of GCSF ameliorates learning and memory impairments and improves disruptions in dendritic morphology spine density, and mature spines in hippocampal CA1 neurons induced by brain ischemia in rats [30]. In vivo treatment with of GCSF restores long-term depression in hippocampal slices from transgenic the APP/PS1 mouse model of Alzheimer’s disease (AD) [31]. In the most common type of pediatric brain tumor, medulloblastoma (MB), the expression of the GCSF receptor CD144 is found in MB cell lines, patient-derived xenograft (PDX) tumors, and primary patient tumors. CD114+ cells show resistance against cytotoxic chemotherapy and are responsive to stimulation by GCSF [17]. In addition, transcript levels of the *CSF3R* gene have been identified across molecular subgroups of MB [18]. A previous study focusing on human gliomas described widespread RNA and protein expression of GCSF. In addition, proliferation and migration were stimulated by exposure to GCSF in CD114+ glioma cells, whereas GCSF inhibition by a neutralizing antibody impaired cell growth and migration [11]. However, the potential role of *CSF3R*/CD114 as a biomarker in gliomas remains poorly understood and warrants further investigation. In the present study, we describe transcript levels of *CSF3R* in gliomas and their association with patient prognosis as assessed by overall survival (OS).

## 2. Results

### 2.1. CSF3R/CD114 Transcript Levels Are Higher in Different Glioma Types Compared to Non-Tumoral Neural Tissue

**Figure 1:**
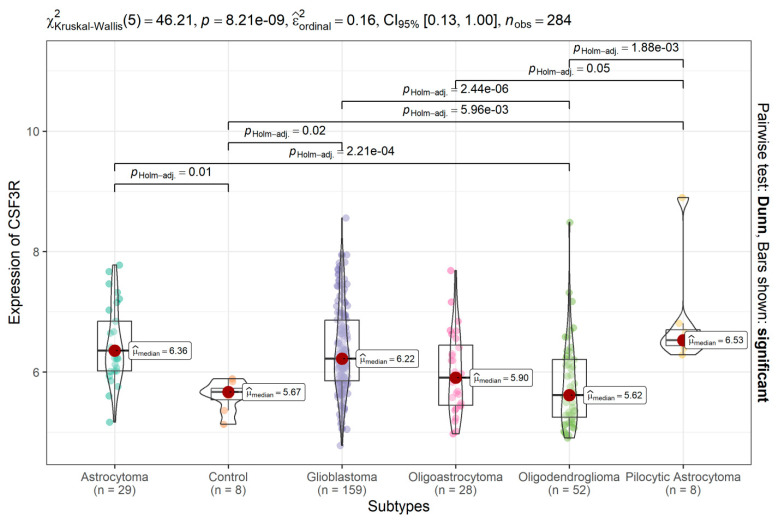
*CSF3R*/CD114 gene expression in different types of glioma and non-tumoral neural tissue. Data were obtained from the French cohort (total dataset n = 284) and analyzed with the R2 Genomics Analysis and Visualization Platform (http://r2.amc.nl). Results are presented in boxplot format as log2-transformed signal intensity. Glioma tumor samples were classified as astrocytoma, pilocytic astrocytoma, oligoastrocytoma, oligodendroglioma, and glioblastoma.; *p* values for comparisons are indicated in the figure.

In comparison to non-tumoral neural tissue (n = 8), significantly higher levels of *CSF3R* transcripts were observed in astrocytoma, pilocytic astrocytoma, and glioblastoma (all *p*s < 0.001; Figure 1; Table 1), whereas oligoastrocytoma and oligodendroglioma did not show significant differences in the French cohort.

### 2.2. Higher Gene Expression of CSF3R/CD114 Is Associated with Poorer Patient Outcome in GBM

**Figure 2:**
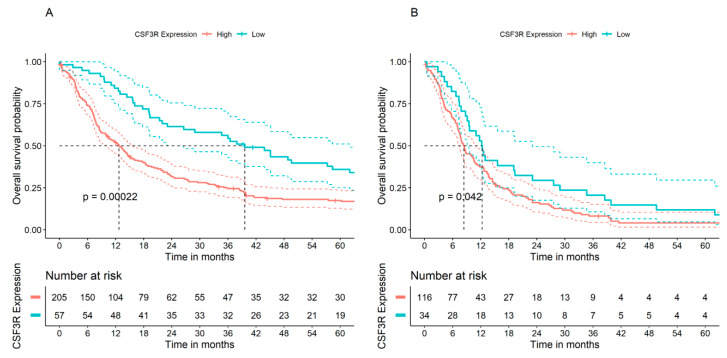
*CSF3R*/CD114 gene expression and OS in patients with glioma. Results are shown for (**A**) all gliomas pooled together (n = 266) or (**B**) GBM patients only (n = 150). Data were obtained from the French cohort. Patient OS was measured from the day of diagnosis until death or date of last follow-up, and calculated using the Kaplan–Meier estimate, with median values and long-rank statistics; *p* values are indicated in the figure.

OS was analyzed using 266 glioma samples from the French dataset. Patients were divided into two groups (low or high expression) based on the expression level of the *CSF3R*/CD114 gene. Analysis of glioma patient OS in relation to *CSF3R*/CD114 transcript levels in tumors showed that, when all glioma types were pooled together, higher *CSF3R*/CD114 expression was significantly associated with a poorer prognosis as assessed by shorter OS (Figure 2A). We then assessed each glioma type, grouping grade II and III samples together due to the limited number of samples in each subgroup. A significant association between high *CSF3R* mRNA levels and shorter OS was observed in patients with GBM (n = 150; *p* < 0.05; Figure 2B).

### 2.3. CSF3R/CD114 Transcript Levels and OS in Patients Bearing IDH-Mutated versus IDH Wild-Type GBM Tumors

**Figure 3:**
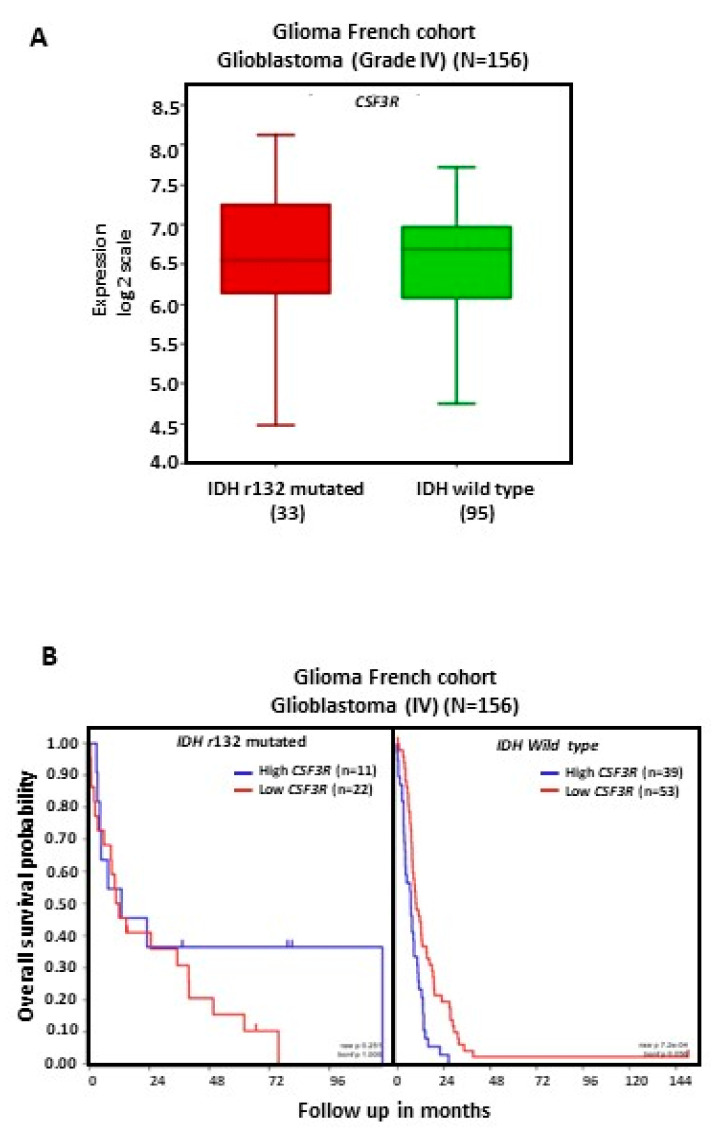
*CSF3R*/CD114 expression and OS in patients bearing *IDH*-mutated versus *IDH* wild-type GBM tumors. Data were obtained from the French cohort. (**A**) Results for gene expression are presented in boxplot format as log2-transformed signal intensity. (**B**) Patient OS was measured from the day of diagnosis until death or date of last follow-up and calculated using the Kaplan–Meier estimate.

Patients were divided into *IDH*-mutated and *IDH* wild-type tumor groups, and then patients within each group were also classified according to high or low *CSF3R* expression levels. There was no significant difference in *CSF3R* mRNA expression between patients with *IDH*-mutated versus wild-type GBM tumors (Figure 3A). There were no significant differences in OS between patients with low- or high-expressing tumors within the *IDH*-mutated or *IDH* wild-type groups. Patients with wild-type *IDH* showed an apparent reduction in OS regardless of *CSF3R* levels (Figure 3B).

### 2.4. CSF3R/CD114 Transcript Levels in the Brain Lower Grade Glioma (TCGA-LGG) Cohort

**Figure 4:**
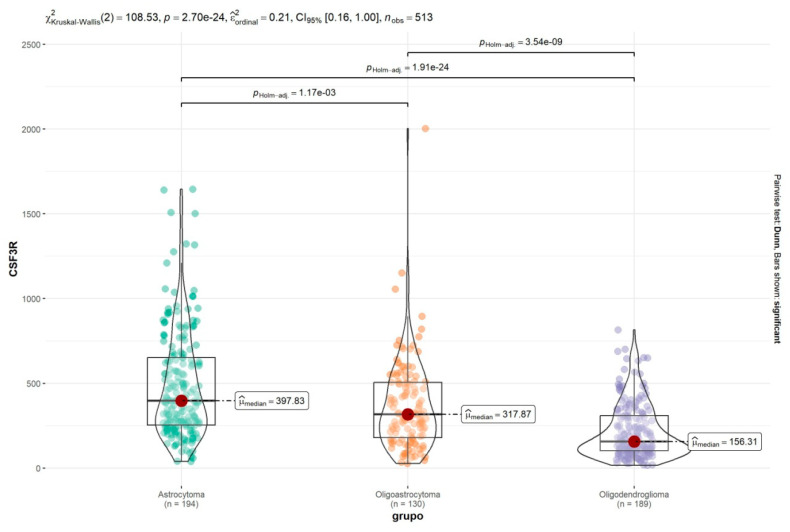
*CSF3R*/CD114 expression in astrocytoma (n = 194), oligoastrocytoma (n = 130), and oligodendroglioma (n = 189) tumors from the TCGA-LGG cohort; *p* values are indicated in the figure.

We went on to analyze *CSF3R*/CD114 expression in The Cancer Genome Atlas (TCGA) Brain Lower Grade Glioma (TCGA-LGG) cohort, which contains 513 glioma samples distributed across tumor types astrocytoma, oligoastrocytoma, and oligodendroglioma. Consistently with the data obtained with the French cohort, astrocytoma tumors displayed the highest gene expression of *CSF3R* (Figure 4). 

### 2.5. Higher CSF3R/CD114 Gene Expression Is Associated with Shorter Patient OS in Glioma Tumors from the TCGA-LGG Cohort

**Figure 5:**
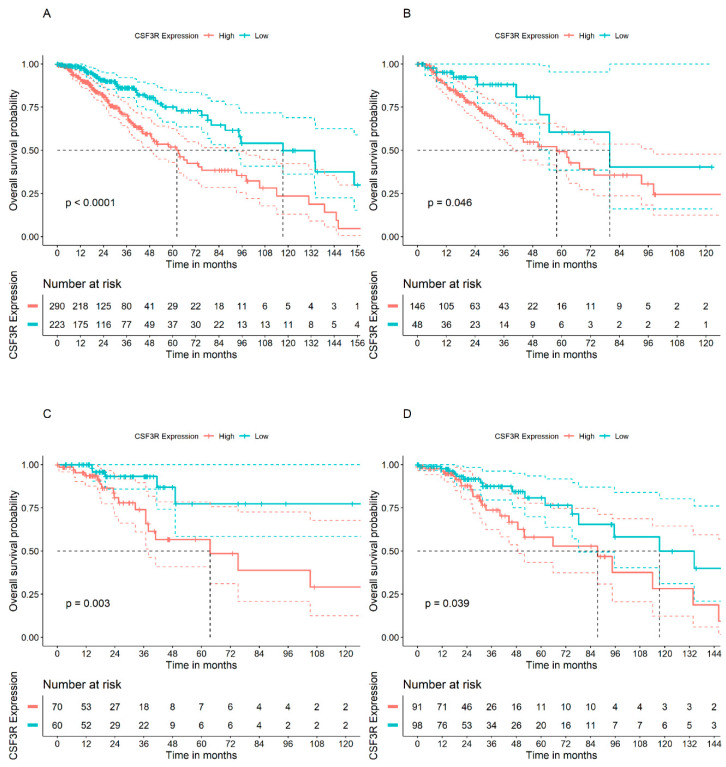
*CSF3R*/CD114 gene expression and OS of patients in the TCGA-LGG cohort. (**A**) glioma types polled together, (**B**) astrocytoma (n = 194), (**C**) oligoastrocytoma (n = 130), and (**D**) oligodendroglioma (n = 189).

In glioma tumors from the TCGA-LGG dataset, a poorer patient prognosis as assessed by shorter OS was significantly associated with high *CSF3R*/CD114 expression when all tumor types were polled together (Figure 5A), as well as when astrocytoma (n = 194), oligoastrocytoma (n = 130), and oligodendroglioma (n = 189) tumors were analyzed separately (Figure 5B–D).

## 3. Discussion

GCSF is a cytokine encoded by the *CSF3* gene that acts as a hematopoietic growth factor regulating the function of granulocytic precursors and neutrophils. GCSF actions are mediated by activation of its receptor, named GCSFR or CD114. Recombinant human GCSF is clinically used to prevent neutropenia, due to its effects on neutrophil mobilization and maturation [32]. GCSF/CD114 signaling has also been investigated as a modulator of neuronal survival, synaptic plasticity [6,28,29,30,31], and cancer [7,10,11,12,13,14,15,16,17,18,19,20]. Specifically, CD114 has been put forward as a marker to identify CSC subpopulations associated with tumorigenicity, metastasis, and resistance to treatment [7,9,10].

In epithelial skin tumors, the presence of CD114 is significantly higher compared to normal skin, Bowen’s disease, or actinic keratosis, and was associated with carcinogenesis. However, no association between the protein expression of CD114 and patient mortality was found [13]. Similarly, different levels of *CSF3R* transcripts occur among different tumor subgroups and subtypes of MB, but no significant association with patient survival was established [18]. A previous study in glioma analyzed the RNA and protein expression of GCSF and CD114 in a set of 22 human gliomas (WHO grade II, III, and IV) and cell cultures derived from these tumors. Although the expression of GCSF and CD114, as well as that of granulocyte macrophage colony-stimulating factor (GMCSF) and its receptor, was found in all glioma tumors and cell cultures, the coexpression of both factors and their receptors was selectively observed in grade IV tumors (GBMs), and thus, the expression correlates with advanced tumor stage [11]. The present transcript analyses indicate that a significant association between high *CSF3R* mRNA levels and poorer prognosis measured by shorter OS was found in patients with gliomas. These early in silico findings suggest that further experimental studies should characterize the effects of GCSF/CD114 inhibition in experimental GBM models.

We did not find a significant impact of *CSF3R* mRNA expression on OS when GBM patients were divided according to *IDH* status (mutated versus wild-type), possibly because of the limited number of samples available in each subgroup. Also, there was no significant difference in *CSF3R* levels between mutated or *IDH* wild-type GBM tumors. Some *IDH1* mutations are considered prognostic markers, with patients bearing mutated tumors showing improved OS [33] Consistently with these data, we found an apparently poorer OS in patients with wild-type *IDH* GBM. One study reported that in mice bearing mutated *IDH* GBM, GCSF is secreted by GBM CSCs, and blocking GCSF accelerates tumor progression by acting on tumor-infiltrating myeloid cells [34].

STAT3 is involved in mediating the cellular effects of CD114 activation. Several studies have indicated that STAT3 is an oncogene in GBM. The activation of STAT3 is associated with shorter OS and progression-free survival in patients with GBM [35], and STAT3 is required for the maintenance of a CSC phenotype in GBM cells [36]. STAT3 may have a dual role in GBM, either promoting or suppressing GBM tumor progression [37,38]. Future experiments should investigate the role of STAT3 downstream of CD114 in different glioma types.

## 4. Materials and Methods

### 4.1. Gene Expression, and Tumor and Patient Data

*CSF3R* mRNA expression levels were normalized using the R2 Genomics Analysis and Visualization Platform (http://r2.amc.nl). Data were obtained from the French cohort (total n = 284; Gene Expression Omnibus—GEO ID: GSE16011; https://www.ncbi.nlm.nih.gov/geo/query/acc.cgi?acc=gse16011, website accessed on 31 January 2024), which included samples from different primary glioma types as well as non-tumoral neural tissue as a control [26,39,40]. Patient characteristics are summarized in Table 1. Tumors were classified into different types according to data available in the dataset.

Normalization of raw microarray data was performed using the Robust Multichip Average (RMA) method, and quality control was conducted through Affy Bioconductor/R. We also used data from the TCGA-LGG cohort (total n = 513). The already processed and normalized expression data were obtained from the cBioPortal. Characteristics of patients in the TCGA-LGG dataset are shown in Table 2.

### 4.2. Statistical Analysis

Clinical information of patients in the French cohort was obtained through the ‘geoquery’ package and data described by Gravendeel et al. [39]. Clinical information of patients in the TCGA-LGG cohort was acquired through the cBioPortal. To investigate differences between glioma tumor types and control neural tissue in the French cohort, we used the Wilcoxon test and the Dunn test to perform specific comparisons among tissue types. Assessment of statistical significance was conducted through the Holm-adjusted *p*-value test. Analyses were carried out using the ‘ggstatsplot’ package.

To examine associations between gene expression and patient OS in the French cohort, 8 control samples and additional 12 glioma samples that lacked information about patient status (‘alive’ or ‘dead’) were excluded from the analysis, resulting in a total of 266 samples. To classify patients into high and low *CSF3R* gene expression groups, we used the ‘Survminer’ package with ‘minprop = 0.2’. Patients within specific *IDH*-mutated and *IDH* wild-type groups were also stratified based on high and low *CSF3R* expression levels. Survival analysis was conducted using the ‘Survival’ package. Patient overall survival (OS) was measured from the day of diagnosis until death or date of last follow-up. OS was calculated using the Kaplan–Meier estimate.

The R2 Genomics Analysis and Visualization Platform (http://r2.amc.nl) was used to compare patients with *IDH*-mutated versus *IDH* wild-type GBM tumors from the French cohort. Welch’s ANOVA was carried out for these comparisons, with *p* values < 0.01 considered to indicate statistical significance. Kaplan–Meier survival curves were also estimated for *IDH*-mutated and *IDH* wild-type groups to assess patient OS.

## 5. Conclusions

The main novel finding of the present study, obtained by analyzing public glioma and neural tissue data, is the association of poorer patient outcome assessed by a reduction in OS in patients with high *CSF3R*/CD114 mRNA expression in different types of glioma tumors. Further studies should explore the role of CD114 in glioma tumor cell lines, primary tumors, and tumor microenvironment to increase our understanding of the role of GCSF and similar growth factors in brain cancer progression.

## Figures and Tables

**Table 1:** Summary of characteristics of patients from the French cohort selected for survival analysis.

		Glioma Type				
Characteristics		Astrocytoma	Glioblastoma	Oligoastrocytoma	Oligodendroglioma	Pilocytic Astrocytoma
Total number of samples		28	153	26	51	8
Mean age (years)		42.54	53.84	48.02	49.16	25.49
Gender	Male	20	103	18	31	4
	Female	8	50	8	20	4
Mean overall survival (OS, months)		32.57	16.25	45.60	70.99	74.72
Status	Alive	4	3	2	7	6
	Dead	23	143	24	43	0
Grade	Grade_II	12	0	3	7	0
	Grade_III	16	0	23	44	0

**Table 2:** Summary of characteristics of patients in the TCGA-LGG cohort.

		Glioma Type		
Characteristics		Astrocytoma	Oligoastrocytoma	Oligodendroglioma
Total number of samples		194	130	189
Mean age (years)		41.8	40.96	45.39
Gender	Male	108	72	105
	Female	86	58	84
Mean overall survival (OS, months)		28.93	30.5	35.28
Status	Alive	136	105	147
	Dead	58	25	42
Grade	Grade_II	63	74	111
	Grade_III	131	55	78

## Data Availability

The data presented in this study are available upon request from the corresponding author.
